# Editorial: Reviews in: multiple sclerosis and neuroimmunology

**DOI:** 10.3389/fneur.2026.1746702

**Published:** 2026-02-18

**Authors:** Omid Mirmosayyeb

**Affiliations:** Department of Neurological Sciences, University of Vermont, Burlington, VT, United States

**Keywords:** encephalitis, multiple sclerosis, nervous system, neuroimmunology, review

Neuroimmunological disorders are immune-mediated cascades that affect central and peripheral nervous systems and may lead to inflammation, demyelination, or axonal damage (1). They are classified into a wide spectrum of disorders based on their pathophysiology and autoantibodies produced. Multiple sclerosis (MS), neuromyelitis optica spectrum disorder (NMOSD), myelin oligodendrocyte glycoprotein antibody-associated disease (MOGAD), and autoimmune encephalitis affect the central nervous system (CNS) (2, 3). Guillain-Barré syndrome (GBS) and chronic inflammatory demyelinating polyneuropathy (CIDP) are immune-mediated peripheral neuropathies that affect the peripheral nervous system (PNS) (4).

Neuroimmunological disorders may manifest with various symptoms, including but not limited to sensory or motor deficits, visual disturbances, cognitive impairment, and autonomic dysfunction that reflect the associated neural structures targeted by the immune system (5, 6). Diagnostic workup includes physical examination, imaging modalities, and serum or cerebrospinal fluid (CSF) biomarkers (7, 8). Management most commonly consists of high-dose corticosteroids, intravenous immunoglobulin (IVIG), plasma exchange, immunomodulator agents, or monoclonal antibodies (9, 10). Symptomatic and supportive treatments like rehabilitation and physical therapy are also applicable (6). Early diagnosis and initiation of treatment improve prognosis. However, extensive axonal injury increases the chance of relapses, disability, or residual neurological damage (11, 12).

Current neuroimmunology research focuses on studying the mechanisms underlying the breakdown of immune tolerance in the CNS and PNS which leads to inflammatory, demyelinating, and neurodegenerative sequels (13, 14). One of the most critical aspects is identifying biomarkers that specify neuroaxonal damage and disease activity (15). Recent progress in single-cell and multi-omics technologies and high-resolution neuroimaging techniques improves the understanding of interactions between immune and glial cells, blood-brain-barrier integrity, and neuroinflammation (16, 17). Therapeutic research focuses on precision immunotherapies, such as B-cell depletion, complement modulation, and new approaches like chimeric antigen receptor (CAR) T-cell therapy (8, 18, 19).

The current issue includes reviews that consolidate the original studies on different challenges in neuroimmunology research. They include topics from mechanisms of neuroinflammation such as fibrosis in impaired neural repair in MS, microglia in pathogenesis of MS, and pathogenesis of anti-NMDAR encephalitis to therapeutic topics, for example DMTs in cognitive impairment in MS, DMTs in pediatric onset MS, hematologic implications of DMTs, photobiomodulation in neuroprotection, as well as epidemiology of inflammatory activity in primary progressive MS (PPMS), ethnic characteristics in MS, and economic burden of MOGAD. The articles in this issue present a comprehensive overview of available literature on the immunopathological mechanisms underlying disease initiation and progression, neural repair, and therapeutic strategies. Further studies that integrate the immunological understanding with translational approaches are warranted to achieve personalized management in neuroimmunological disorders. In the following section, the specific scope and key contributions of each review included in this issue has been outlined to highlight how these diverse perspectives advance the field of neuroimmunology:

One review focused on the role of neurofibrosis in MS and highlighted how extracellular matrix remodeling contribute to impaired neural repair and disease progression (Lozinski et al.). Another paper reviewed the contribution of microglia to chronic neuroinflammation and neurodegeneration in MS and emphasized emerging therapeutic strategies aimed at modulating microglial activity (Vermersch et al.). A mini-review addressed blood–brain barrier dysfunction in anti-NMDAR encephalitis and demonstrated how compromised barrier integrity facilitates pathogenic immune infiltration into the central nervous system (Gong et al.). One clinical review evaluated the effects of disease-modifying therapies on cognitive outcomes in MS and suggested that selected treatments may stabilize or improve cognitive performance (Kania et al.). Another review on the immunological rationale for induction therapy with alemtuzumab in pediatric-onset MS highlighted age-dependent disease mechanisms and immune reconstitution dynamics (Puthenparampil et al.). A systematic review synthesized evidence on hematological adverse events associated with disease-modifying therapies, emphasizing the importance of long-term hematological monitoring (Scavone et al.). One meta-analysis explored the effects of photobiomodulation in experimental autoimmune encephalomyelitis and reported consistent reductions in neuroinflammation and disease severity in preclinical models (Ahmed). A further systematic review and meta-analysis demonstrated that inflammatory disease activity remains prevalent in PPMS, with younger age and longer radiological follow-up emerging as key predictors (Blok et al.). A perspective review synthesized clinical and translational evidence to propose that increased humoral immune activity, particularly B cell responses, may be a key biological contributor to the disproportionately severe MS course observed in Black/African American and Hispanic/Latinx patients, and underscored the urgent need for more immunology-focused studies across ethnic groups (Telesford et al.). Finally, one narrative review evaluated the economic burden of MOGAD and analogous autoimmune neurological conditions and demonstrated that they impose substantial direct and indirect costs on patients and healthcare systems, with disease severity, relapses, disability, and need for care identified as major cost drivers (Lee et al.).
